# Cells Deficient in the Fanconi Anemia Protein FANCD2 are Hypersensitive to the Cytotoxicity and DNA Damage Induced by Coffee and Caffeic Acid

**DOI:** 10.3390/toxins8070211

**Published:** 2016-07-08

**Authors:** Estefanía Burgos-Morón, José Manuel Calderón-Montaño, Manuel Luis Orta, Emilio Guillén-Mancina, Santiago Mateos, Miguel López-Lázaro

**Affiliations:** 1 Department of Pharmacology, Faculty of Pharmacy, University of Seville, Profesor García González 2, 41012 Seville, Spain; eburgos1@us.es (E.B.-M.); jose.calderon@cabimer.es (J.M.C.-M.); eguillen@us.es (E.G.-M.); 2 Department of Molecular Biology, Centro Andaluz de Biología Molecular y Medicina Regenerativa, University of Seville, Avda. Americo Vespucio s/n., 41092 Seville, Spain; 3 Department of Cell Biology, Faculty of Biology, University of Seville, Avda. Reina Mercedes s/n., 41012 Seville, Spain; morta2@us.es (M.L.O.); smateos@us.es (S.M.)

**Keywords:** coffee, caffeic acid, cancer, DNA damage, carcinogenesis, FANCD2, Fanconi anemia

## Abstract

Epidemiological studies have found a positive association between coffee consumption and a lower risk of cardiovascular disorders, some cancers, diabetes, Parkinson and Alzheimer disease. Coffee consumption, however, has also been linked to an increased risk of developing some types of cancer, including bladder cancer in adults and leukemia in children of mothers who drink coffee during pregnancy. Since cancer is driven by the accumulation of DNA alterations, the ability of the coffee constituent caffeic acid to induce DNA damage in cells may play a role in the carcinogenic potential of this beverage. This carcinogenic potential may be exacerbated in cells with DNA repair defects. People with the genetic disease Fanconi Anemia have DNA repair deficiencies and are predisposed to several cancers, particularly acute myeloid leukemia. Defects in the DNA repair protein Fanconi Anemia D2 (FANCD2) also play an important role in the development of a variety of cancers (e.g., bladder cancer) in people without this genetic disease. This communication shows that cells deficient in FANCD2 are hypersensitive to the cytotoxicity (clonogenic assay) and DNA damage (γ-H2AX and 53BP1 focus assay) induced by caffeic acid and by a commercial lyophilized coffee extract. These data suggest that people with Fanconi Anemia, or healthy people who develop sporadic mutations in FANCD2, may be hypersensitive to the carcinogenic activity of coffee.

## 1. Introduction

Coffee, one of the most widely consumed beverages in the world, can affect human health. Observational studies suggest that coffee consumption may lower the risk of developing diabetes, cardiovascular disorders, cirrhosis, and degenerative disorders such as Parkinson and Alzheimer disease [1,2]. Coffee consumption has also been associated with a decreased risk of total mortality; coffee drinkers had a lower risk of death from heart disease, chronic respiratory diseases, diabetes, pneumonia and influenza, and intentional self-harm [3]. No significant association between coffee consumption and total cancer mortality was found, however [3]. The effect of coffee consumption on the risk of cancer is inconclusive; some studies indicate that coffee may reduce the risk of some types of cancers [4,5,6], while others suggest that it may increase the risk of developing the disease [6,7]. According to the International Agency for Research on Cancer (IARC), coffee is classified as possibly carcinogenic to the human urinary bladder (IARC, Vol. 51). Several recent epidemiological studies have also revealed that maternal consumption of coffee during pregnancy may be associated with childhood leukemia [8,9,10,11]. A recent meta-analysis found that maternal coffee consumption during pregnancy significantly increased the risk of childhood acute lymphoblastic leukemia (ALL) and acute myeloid leukemia (AML) in a dose-response manner [11].

Caffeic acid is present in coffee in low amounts, but it is an important metabolite found in plasma and urine after coffee consumption [1,12]. In coffee, caffeic acid is typically bound to quinic acid to form an ester called 5-caffeoylquinic acid or chlorogenic acid [13]. Some studies have shown that caffeic acid possesses antioxidant and anti-genotoxic activities [14,15,16,17,18,19]. However, other investigations have revealed that this polyphenol generates reactive oxygen species (ROS) and produces carcinogenic effects [20,21,22,23,24,25,26]. In vivo studies have also found that diets containing caffeic acid induced tumors in animals [2]. Caffeic acid is therefore classified as possibly carcinogenic to humans (IARC, Vol. 56). This carcinogenic activity may be due to its ability to induce DNA damage [21,22,23], probably through a pro-oxidant mechanism [22,24,25,26].

Cancer is a disease caused by the accumulation of DNA alterations in our cells [27,28,29,30]. When our cells suffer DNA alterations, the DNA damage response machinery activates a variety of mechanisms to repair the damage [3]. These mechanisms are necessary for maintaining the integrity of the DNA, and therefore provide a fundamental biological barrier against carcinogenesis. Mutations in DNA repair genes result in the accumulation of cellular DNA damage and in predisposition to cancer. Some people are born with defects in genes involved in the DNA damage response machinery. People without germline mutations in DNA repair genes can also acquire them during a possible carcinogenic process. People with these mutations are particularly sensitive to the carcinogenic activity of compounds that induce types of DNA damage requiring these genes for repair.

The Fanconi anemia (FA) pathway plays an important role in the repair of several types of DNA damage, including interstrand crosslink, replication fork stalling and double strand breaks. The FA protein Fanconi Anemia D2 (FANCD2) is essential for proper functioning of this pathway; this protein is considered a surrogate marker for FA network activation. Mutations in a cluster of proteins of this pathway cause Fanconi Anemia, a rare autosomal recessive genetic disease characterized by bone marrow failure, congenital abnormalities, genomic instability and predisposition to several types of cancer, particularly acute myeloid leukemia [31,32,33]. Defects in proteins implicated in this DNA repair pathway has been described in several kinds of sporadic cancers, including bladder cancer and acute myeloid leukemia [34,35,36,37]. Deficiency in this DNA repair pathway makes cells hypersensitive to the cytotoxicity and DNA-damaging activities of a variety of agents, including ROS [38,39,40].

Since caffeic acid induces ROS-mediated DNA damage, and since the FA pathway participates in the repair of this type of DNA damage, we hypothesized that cells deficient in this pathway would be more susceptible than normal cells to the DNA-damaging effect of this dietary phytochemical. We report that human cells deficient in FANCD2 are hypersensitive to the cytotoxicity (clonogenic assay) and DNA damage (γ-H2AX and 53BP1 focus assay) induced by caffeic acid and by a commercial lyophilized coffee extract, and discuss the possible relevance of these results.

## 2. Results

### 2.1. Cells Deficient in FANCD2 Are Hypersensitive to the Cytotoxicity of Coffee and Caffeic Acid

**Figure 1:**
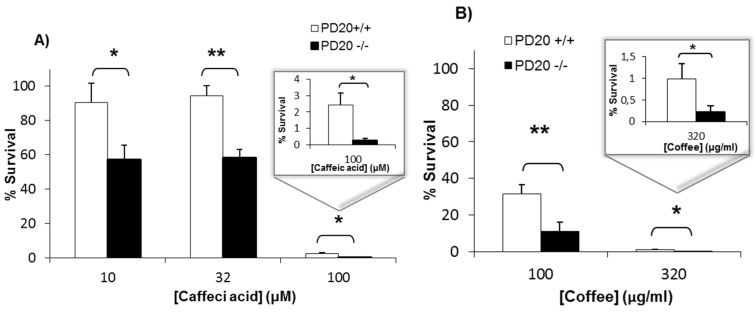
Cells deficient in Fanconi Anemia D2 (FANCD2) are hypersensitive to the cytotoxicity of caffeic acid (**A**) and a commercial lyophilized coffee extract (**B**). Parental PD20 cells with functional FANCD2 (PD20+/+) and PD20 cells lacking FANCD2 (PD20−/−) were treated with caffeic acid or coffee for 4 h. Then, the cells were allowed to form colonies in drug-free medium for 7 days, and the percentage of cell survival with respect to untreated cells was determined with the clonogenic assay. Data show the mean and standard deviation (SD) from at least 3 independent experiments. For statistical analysis, the *t*-test (paired, two-tailed) was used (* *p* < 0.05, ** *p* < 0.01).

Cells lacking the FA protein FANCD2 (PD20−/−) and cells complemented with FANCD2 (PD20+/+) were exposed for 4 h to caffeic acid and to a commercial lyophilized coffee extract. After 7 days of recovery in drug-free medium, cell survival was determined with the clonogenic assay. Figure 1 shows that the survival of cells lacking FANCD2 was significantly lower than that of proficient cells when exposed to several concentrations of caffeic acid (A) and coffee (B). This means that the cellular toxicity of coffee and caffeic acid is increased in cells lacking the DNA repair protein FANCD2. 

### 2.2. Cells Deficient in FANCD2 Are Hypersensitive to the DNA Damage Induced by Coffee and Caffeic Acid

**Figure 2:**
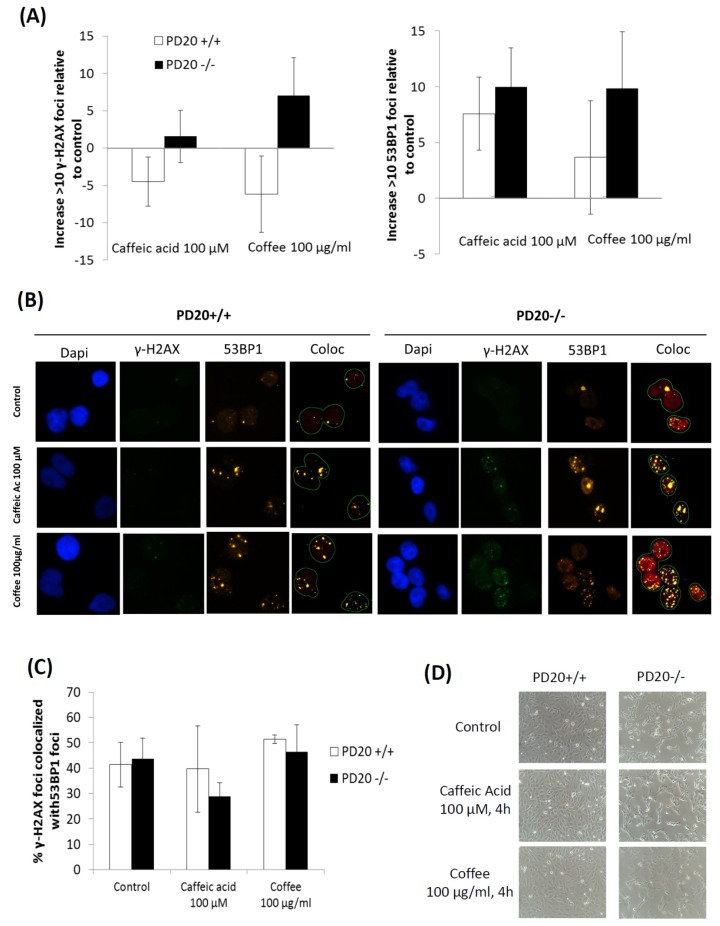
Cells deficient in FANCD2 (PD20−/−) are more sensitive than non-deficient cells (PD20+/+) to the DNA damage induced by a commercial lyophilized coffee extract and by caffeic acid. Cells were exposed for 4 h to caffeic acid 100 μM or coffee 100 μg/mL, and the levels of γ-H2AX and 53BP1 foci were measured with the Immunofluorescence focus assay. In (**A**), quantification of nuclear foci is presented. Data show the mean and standard deviation (SD) from at least 3 independent experiments; *p* > 0.05 (*t*-test, paired, two-tailed). Representative micrographs are shown in (**B**), where γ-H2AX foci appear as green spots, 53BP1 foci appear as orange spots and DAPI (4′,6-diamidino-2-phenylindole)-stained nucleus appear in blue. γ-H2AX foci colocalized with 53BP1 appear as yellow spots. Pictures were taken with an Olympus BX 61 microscope at 40-fold magnification (Figure 2B shows the part of the pictures that contained cells). In (**C**), the percentage of γ-H2AX foci colocalized with 53BP1 is represented. In (**D**), representative photographs of control cells and cells exposed for 4 h to caffeic acid 100 μM or coffee 100 μg/mL are shown.

We used the immunofluorescence focus assay to measure the levels of DNA damage in FANCD2 deficient and proficient cells exposed to coffee and caffeic acid. We used specific antibodies to determine the levels of γ-H2AX and 53BP1 foci. An increase in the cellular levels of γ-H2AX foci is associated with the formation of double strand breaks (DSBs) in the DNA, but also with the formation of other types of DNA damage; γ-H2AX can therefore be considered as a marker of general DNA damage [4,41]. Formation of γ-H2AX foci is associated with recruitment of p53-binding protein 1 (53BP1), a regulator of the cellular response to DNA double-strand breaks. Therefore, the presence of 53BP1 foci is a specific marker of DSBs. Figure 2 shows that cells lacking FANCD2 developed higher levels of γ-H2AX foci and 53BP1 foci than non-deficient cells when exposed to coffee and caffeic acid.

## 3. Discussion

Coffee consumption may increase the risk of developing some types of cancer, including bladder cancer and childhood leukemia. Caffeic acid, a phenolic compound found in plasma and urine after coffee consumption, may contribute to the carcinogenic potential of coffee. Although the effect of coffee consumption on the risk of cancer is inconclusive, the International Agency for Research on Cancer has classified both coffee and caffeic acid as possibly carcinogenic to humans.

The ability of caffeic acid to induce DNA damage in cells [22,23,24,25,26] may play a key role in the carcinogenic potential of caffeic acid and coffee. It is well-known that dividing cells are more susceptible to DNA-damaging agents than non-dividing cells. Because cells divide actively during embryonic and fetal development, coffee consumption could be particularly carcinogenic during pregnancy. In addition, the carcinogenic potential of coffee would be higher in cells deficient in particular DNA repair proteins. Figure 1 shows that cells deficient in FANCD2, a critical DNA repair protein of the Fanconi Anemia pathway, are hypersensitive to the cytotoxicity of caffeic acid and coffee. This suggests that coffee and caffeic acid cause more DNA damage in cells deficient in this DNA repair protein. Figure 2 shows that cells lacking FANCD2 developed higher levels γ-H2AX foci than non-deficient cells when exposed to coffee and caffeic acid. This suggests that the DNA-damaging effects of coffee and caffeic acid are increased in cells with defects in the DNA repair protein FANCD2. The levels of 53BP1 foci were also increased in cells lacking FANCD2, therefore indicating that coffee and caffeic acid induce more DSBs in cells deficient in this DNA repair protein. Together, this data indicate that cells deficient in FANCD2 are hypersensitive to the cytotoxicity and DNA-damaging activities of caffeic acid and coffee, and suggest that people with mutations in FANCD2 may be hypersensitive to their carcinogenic activity.

The bioavailability of caffeic acid in humans is relatively high. The plasma and urinary levels of caffeic acid in humans after coffee consumption are typically in the nanomolar and low micromolar range [12,42,43]. For example, a study showed that the peak concentration of caffeic acid in the plasma of ten healthy adults was 1.1 ± 0.9 μM; its concentration in urine was highly variable, ranging from 0.07 to 9.43 μM [5]. Figure 2 shows that the concentration of caffeic acid required to detect DNA damage in cells is high (100 μM), probably because the sensitivity of the immunofluorescence focus assay is relatively low. But Figure 1 shows that, when treated with caffeic acid 10 μM, the survival of cells deficient in the DNA repair protein FANCD2 is approximately 60% of that of untreated cells. This suggests that caffeic acid 10 μM induces cytotoxic levels of DNA damage, and that non-cytotoxic levels of DNA damage probably occur at lower concentrations; these concentrations are possibly similar to those achieved in plasma and urine after coffee consumption. 

The high concentrations of caffeic acid detected in the urine of some healthy volunteers after coffee consumption [6] indicate that this phytochemical can be accumulated in the urinary bladder. This may explain why coffee is classified as possibly carcinogenic to the human urinary bladder (IARC, Vol. 51). FANCD2 deficiencies have been described in bladder cancer [34,35,36]. Together, this supports the idea that coffee and caffeic acid may increase the risk of bladder cancer, particularly in people with germline or sporadic mutations in the DNA repair protein FANCD2. 

The carcinogenic activity of coffee may be mediated not only by the pro-oxidant activity of caffeic acid [22,24,25,26], but also by other pro-oxidant coffee constituents. Several coffee constituents, including chlorogenic acid and hydroquinone, are known to generate hydrogen peroxide [44,45,46,47], and hydrogen peroxide induces DNA damage and plays a key role in cancer development [48]. The cytotoxicity and DNA-damaging activities of hydrogen peroxide and other ROS are higher in cells deficient in DNA repair proteins of the Anemia Fanconi pathway [38,39,40]. This suggests that the carcinogenic potential of coffee is mediated by the ability of caffeic acid and other coffee constituents to induce pro-oxidant DNA damage, and that this carcinogenic potential is higher in cells lacking DNA repair proteins of the Anemia Fanconi pathway. It is important to clarify that some polyphenols can both prevent and induce oxidative DNA damage, mainly depending on their concentration. At low concentrations, they can reduce the levels of ROS and prevent DNA damage. At higher concentrations, however, some polyphenols (particularly those containing catechol or pyrogallol moieties in their structure, such as caffeic acid) can generate hydrogen peroxide by an autoxidation mechanism. This mechanism involves the oxidation of polyphenols to semiquinones in a process in which oxygen is reduced to superoxide anion and hydrogen peroxide. Through the Fenton reaction, hydrogen peroxide produces hydroxyl radicals that cause oxidative DNA damage [49,50].

In summary, although the effect of coffee consumption on the risk of cancer is inconclusive, some studies indicate that coffee may increase the risk of developing some cancers [6,7]. Here we report that cells deficient in the DNA repair protein FANCD2 are hypersensitive to the cytotoxic and DNA-damaging activities of a commercial lyophilized coffee extract and caffeic acid (an important metabolite found in plasma and urine after coffee consumption). These data suggest that people with Fanconi Anemia, or healthy people who develop sporadic mutations in FANCD2, may be hypersensitive to the carcinogenic activity of coffee.

## 4. Materials and Methods

### 4.1. Chemicals and Cell Lines

Caffeic acid was purchased from Sigma (≥98.0%, Sigma-Aldrich, St. Louis, MO, USA). A commercial lyophilized coffee extract (NESCAFÉ^®^ Classic, Barcelona, Spain) was used. In all experiments, caffeic acid and the coffee extract were tested individually, not mixed together. The human Fanconi deficient (PD20 FANCD2−/−) and proficient (PD20 FANCD2−/− complemented with FANCD2) cells were kindly provided by Dr. Thomas Helleday and by Dr. Jordi Surrallés Calonge. Cells were maintained in DMEM supplemented with 2 mM glutamine, 50 μg/mL penicillin, 50 μg/mL streptomycin and 20% fetal bovine serum. Cells were cultured at 37 °C in a humidified atmosphere containing 5% CO_2_. 

### 4.2. Clonogenic Assay

Cell survival was measured with the clonogenic assay. Cells were plated at low density onto 6 cm Petri dishes. Cells were treated with caffeic acid or coffee for 4 h; then drugs were removed and fresh media was added to allow the cells to grow for 7 days. Colonies were stained with methylene blue prepared in methanol (4 g/L). Surviving colonies made up 50 cells per colony were counted and the data were corrected according to cloning efficiencies of control cells.

### 4.3. Immunofluorescence γ-H2AX and 53BP1 Focus Assay

To evaluate DNA damage, detection of γ-H2AX and 53BP1 foci were quantified by immunofluorescence using the focus assay. The γ-H2AX focus assay is based on the ability of double-strand breaks (DSBs) to trigger phosphorylation of histone H2AX on Ser-139, which leads to the formation of nuclear foci that can be visualized with anti-γ-H2AX antibodies. Following the induction of double strand breaks, formation of γ-H2AX foci is associated with recruitment of p53-binding protein 1 (53BP1). Accumulation of 53BP1 can also be visualized as foci with anti-53BP1 antibodies. After treatments, cells were washed three times with PBS, and incubated for 30 s with cold 0.1% Triton-X in PBS to pre-extract soluble proteins. Afterward, cells were fixed with 4% paraformaldehyde in PBS for 10 min at room temperature and washed three times with PBS. After fixation, cells were permeabilized with 0.5% Triton X-100 in PBS for 5 min and then blocked three times with 0.1% Tween 20, 1% BSA in PBS for 5 min each. Cells were then incubated for 1 h with a mouse anti-γ-H2AX monoclonal antibody (Upstate; 1:800 dilution). Cells were washed three times with PBS and blocked three times prior to the incubation with a secondary anti-mouse antibody linked to Alexa Fluor 488 (Invitrogen; 1:500 dilution) for 1 h. Cells were washed with PBS, blocked, and incubated overnight with the rabbit anti-53BP1 primary antibody (Bethyl, 1:1000 dilution). After incubation, cells were washed with PBS, blocked and washed again with PBS as indicated before. DNA was stained with DAPI (4′,6-diamidino-2-phenylindole) and immunofluorescence was observed at 40-fold magnification with an Olympus BX 61 microscope. A total of at least 100 cells/dose were scored, and cells with 10 or more foci were scored as positive [44,51].

## Abbreviations

ALL	acute lymphoblastic leukemia
AML	acute myeloid leukemia
DSBs	double strand breaks
FA	Fanconi anemia
IARC	International Agency for Research on Cancer
ROS	reactive oxygen species
